# Evaluation of the Diagnostic Performance of the Brush/Biopsy Rapid On-Site Evaluation (B-ROSE) in Cases of Bile Duct Stricture: A Prospective, Pilot Study

**DOI:** 10.3390/jcm14176207

**Published:** 2025-09-02

**Authors:** Nao Hattori, Daisuke Uchida, Kei Harada, Ryosuke Sato, Taisuke Obata, Akihiro Matsumi, Kazuya Miyamoto, Hiroyuki Terasawa, Yuki Fujii, Koichiro Tsutsumi, Shigeru Horiguchi, Kazuyuki Matsumoto, Motoyuki Otsuka

**Affiliations:** 1 Department of Gastroenterology, Okayama University Hospital, 2-5-1 Shikata-cho, Kita-ku, Okayama 700-8558, Japan; nhattori1007@gmail.com (N.H.); matsumoto.k@okayama-u.ac.jp (K.M.);; 2 Center for Innovative Clinical Medicine, Okayama University Hospital, 2-5-1 Shikata-cho, Kita-ku, Okayama 700-8558, Japan

**Keywords:** bile duct stricture, ERCP (endoscopic retrograde cholangiopancreatography), rapid on-site evaluation (ROSE), B-ROSE

## Abstract

**Background:** Biliary strictures are diagnosed using endoscopic retrograde cholangiopancreatography (ERCP) with brush cytology and biopsy. However, brush cytology shows a sensitivity of 9–56.1% and a diagnostic accuracy of 43–65.4%, while biopsy demonstrates a sensitivity of 48%. Both methods exhibit high specificity but limited sensitivity. While rapid on-site evaluation (ROSE) is effective in endoscopic ultrasound-guided fine needle aspiration (EUS-FNA), its application in ERCP-obtained samples remains underexplored. **Methods:** This prospective pilot study was conducted at Okayama University Hospital from April 2019 to July 2024. Patients requiring ERCP-guided sampling for bile duct strictures were included. ROSE was applied to brush cytology with up to three additional attempts and to imprint cytology from biopsy samples with up to two attempts. Diagnostic accuracy was assessed based on pathology and clinical course. **Results:** Among 37 patients (median age: 73 years, add range, and male–female ratio: 27:10), 18 had hilar and 19 had distal bile duct strictures. Brush cytology required one, two, or three attempts in twenty-six, six, and five cases, respectively, whereas biopsy required one or two attempts in thirty-five and two cases, respectively. Among the thirty-seven cases, thirty-five were malignant and two were benign. The B-ROSE group showed a sensitivity, specificity, and accuracy of 71.4%, 100.0%, and 73.0%, respectively, compared to lower accuracy in the conventional group, where single brush cytology attempts yielded a sensitivity of 48.6% and an accuracy of 48.6%, and single biopsy attempts showed a sensitivity of 68.6% and an accuracy of 70.3%. **Conclusions:** B-ROSE improves diagnostic accuracy, reduces repeat sampling, and minimizes patient burden in ERCP-based diagnosis of bile duct strictures, making it a valuable addition to current diagnostic protocols.

## 1. Introduction

Biliary strictures are typically diagnosed using a combination of endoscopic retrograde cholangiopancreatography (ERCP) for biliary imaging and fluoroscopy-guided brush cytology, bile cytology, and stricture biopsy. However, brush cytology has reported sensitivity ranging from 9% to 56.1% and specificity from 61% to 100% [1,2,3], with diagnostic accuracy ranging from 43% to 65.4% [1,2]. Biopsy has been reported to have a sensitivity of 48% and specificity of 99% [4]. While both brush cytology and biopsy demonstrate good specificity, their sensitivity remains low. Although peroral cholangioscopy (POCS) allows direct visualization of lesions and facilitates biopsies, particularly for assessing the superficial spread of cancer, its clinical application faces several limitations. The diagnostic accuracy varies considerably across studies, with a reported sensitivity, specificity, and accuracy of 38.1%, 100%, and 60.6% to 87%, respectively [5,6,7,8,9]. Furthermore, POCS is available only at specialized centers and increases the physical burden on patients during endoscopic procedures.

Recent advances in endoscopic ultrasound-guided fine needle aspiration (EUS-FNA) have also demonstrated utility for diagnosing bile duct strictures [10,11,12,13]. However, concerns regarding bile leakage caused by duct puncture and the potential risk of tumor dissemination in malignant cases have raised questions regarding its safety as a first-line diagnostic approach. To improve the diagnostic accuracy, endoscopic nasobiliary drainage (ENBD) tubes can be placed to collect bile specimens over several days. In some cases, ERCP must be repeated for additional sampling [14,15]. While enhancing the diagnostic yield, these approaches increase the physical and procedural burden on patients.

Rapid on-site evaluation (ROSE) has shown promise for improving the diagnostic accuracy of EUS-FNA. However, their application in bile duct brush cytology has not been explored [16]. The use of ROSE for touch imprint cytology (ROSE-TIC) of surgical and biopsy specimens obtained via endoscopy or POCS has also been reported [17,18]. However, bile duct brush cytology and biopsy have not been systematically evaluated in a prospective study.

To address these gaps, we designed a prospective study to evaluate the utility of ROSE in bile duct brush cytology. This study aimed to determine whether ROSE can enhance diagnostic accuracy, reduce the procedural burden, and provide a reliable approach for the diagnosis of bile duct strictures.

## 2. Materials and Methods

### 2.1. Study Design

A prospective pilot study was conducted at Okayama University Hospital from April 2019 to July 2024. The study included patients who underwent ERCP-guided biliary brushing cytology and biopsy with rapid on-site evaluation (ROSE) for biliary strictures during this period.

The eligibility criteria included (1) age ≥18 years, (2) biliary stricture diagnosed using CT or MRI, and (3) a general condition suitable for undergoing ERCP. The exclusion criteria included (1) allergy to contrast media, (2) performance status of 2 or higher, (3) pregnancy or suspected pregnancy, and (4) consent refusal.

Cases where safe insertion of biopsy forceps into the bile duct was deemed not feasible were excluded from enrollment in this study.

This study was approved by the Ethical Review Board of Okayama University Hospital (approved number K1904-011).

### 2.2. Study Procedure

ERCP was performed using a side-viewing endoscope (TJF-260V or TJF-Q290V; Olympus Medical Systems, Tokyo, Japan). After successful cannulation of the bile duct, cholangiography was performed to visualize the stenotic segment. Following endoscopic sphincterotomy (EST), brush cytology and biopsy of the stenotic area of the bile duct were performed. An RX Cytology Brush (Boston Scientific, Hong Kong) was used for brush cytology, and Radial Jaw biopsy forceps (Boston Scientific) were used for touch imprint cytology.

**Figure 1:**
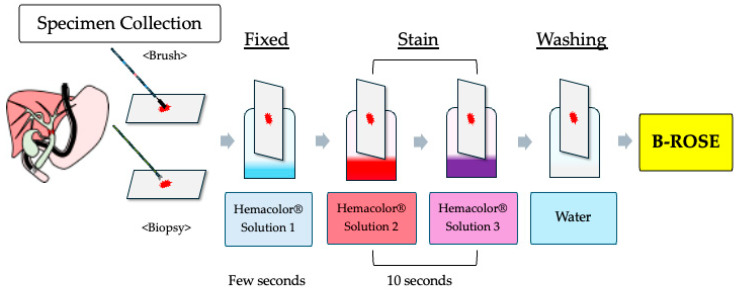
Diagram of the brush/biopsy rapid on-site evaluation (B-ROSE) procedure.

**Figure 2:**
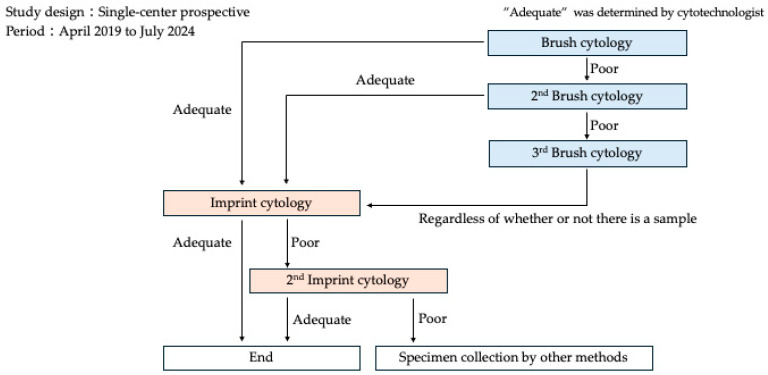
A flowchart of the B-ROSE. B-ROSE was conducted on specimens obtained via brush cytology.

A microscope was installed in the fluoroscopy room where ERCP was performed, and a cytotechnologist was on standby. When specimens were collected during ERCP, the cytotechnologist immediately processed the specimens and performed microscopic evaluation following the procedures described below. The B-ROSE procedure involves several steps to evaluate the adequacy of the specimens obtained during endoscopy (Figure 1). First, specimens were placed on glass slides. For brush cytology, the brush was tapped multiple times with tweezers to transfer the sample onto the glass slide. For imprint cytology, the biopsy specimen was held with tweezers and directly placed on glass slides. The slides were allowed to air-dry. After drying, the slides were sequentially immersed in Hemacolor Solution 1 (Merck, Rahway, NJ, USA) for a few seconds, Hemacolor Solution 2 (Merck) for 10 s, and Hemacolor Solution 3 (Merck) for another 10 s. The excess staining solution was removed by immersing the slides in physiological saline. After staining, the slides were examined under a microscope to determine whether the specimens were appropriate. Once an appropriate specimen was confirmed via B-ROSE, specimen collection was considered successful and biliary drainage was performed if needed. If a specimen was deemed inadequate, alternative collection methods were considered. Specimens were collected during ERCP in accordance with a predefined flowchart to ensure procedural consistency, as depicted in Figure 2. Initially, brush cytology was performed at the site of the bile duct stricture, and the adequacy of the specimen was evaluated using the B-ROSE method. Adequate sampling was defined as the presence of 20 or more nucleated cells per field under 10× objective lens magnification. To avoid prolonged examination time and increased patient burden, malignant versus benign determination was not performed in this B-ROSE method.

For brush cytology, collected samples were tapped onto glass slides multiple times. For biopsy specimens, the harvested tissue was carefully grasped using forceps, gently pressed onto a glass slide, and spread onto another glass slide. The prepared slides were fixed and stained using the Hemacolor rapid staining protocol. Excess staining solution was removed and the slides were air-dried before microscopic examination. A cytotechnologist performed B-ROSE to evaluate specimen adequacy.

In cases where an adequate specimen was collected, a fluoroscopy-guided biopsy was performed. The specimen adequacy was evaluated by cytotechnologists. If a specimen was deemed inadequate, an additional brush cytology was performed, with up to three attempts allowed. On the third attempt, a biopsy was performed regardless of specimen adequacy. Similarly, B-ROSE has been applied to the imprint cytology of fluoroscopy-guided biopsy specimens. The procedure was performed if the specimen was deemed adequate. However, if the specimen was inadequate, additional sampling was performed. When adequacy could not be achieved after two biopsy attempts, alternative specimen collection methods were considered.

If the specimen was determined to be adequate, the procedure was performed until biopsy. If the specimen was inadequate, brush cytology was repeated with up to three attempts allowed. Following brush cytology, a biopsy was performed, and imprint cytology was performed using B-ROSE to confirm specimen adequacy. The procedure was performed if the biopsy specimen was deemed adequate. However, if the specimen remained inadequate, an additional biopsy was conducted with up to two permitted attempts. In cases where no adequate specimens were obtained after two biopsy attempts, alternative methods of specimen collection were explored to ensure accurate diagnostic outcomes.

### 2.3. Outcomes

The primary endpoint was defined as the diagnostic accuracy of ERCP-based cytology and histology for bile duct strictures, whereas the secondary endpoints included the number of specimen collections and incidence of adverse events. Accuracy was defined as the concordance rate between the results of brush cytology or touch imprint cytology of biopsy specimens and the histopathological results of biopsy specimens. For cases where lesions were surgically resected at a later date, accuracy was defined as the concordance rate between the results of brush cytology or touch cytology of biopsy specimens and the pathological results of surgically resected specimens. For cases where a malignant biliary stricture was not diagnosed based on the obtained histopathological results, if a malignant biliary stricture was diagnosed during approximately 6 months of clinical follow-up after the procedure, the final diagnosis was considered to be a malignant biliary stricture.

### 2.4. Statistical Analysis

Categorical variables were expressed as frequencies and percentages. The chi-square test was used to compare categorical variables between groups. McNemar’s test was applied to assess the statistical significance of changes in diagnostic accuracy when comparing the results of single versus repeated sampling procedures (brush cytology and biopsy). Statistical significance was set at *p* < 0.05. All statistical analyses were performed using JMP Student Edition 18.

## 3. Results

### 3.1. Patient Characteristics

Between April 2019 and July 2024, 37 patients with bile duct strictures were enrolled in this study (Table 1). The median age was 73 years (range, 70–78.5 years), and the cohort consisted of 27 males and 10 females. The ECOG-PS distribution was as follows: 27 patients with PS 0, nine with PS 1, one with PS 2, and zero with PS 3. Five patients were receiving antithrombotic medications, which were managed according to established guidelines. The strictures were located in the distal bile duct in 19 patients and in the hilar bile duct in 18 patients. The study population consisted of 27 males (73.0%) and 10 females (27.0%). The chi-square goodness-of-fit test revealed a statistically significant deviation from the expected 1:1 gender ratio (χ^2^ = 7.81, df = 1, *p* = 0.005). The lesions were distributed as follows: distal bile duct in 19 cases (51.4%) and hilar bile duct in 18 cases (48.6%). The chi-square goodness-of-fit test showed no significant deviation from equal distribution (χ^2^ = 0.027, df = 1, *p* = 0.87).

Based on the histological results or clinical follow-up, 35 of the 37 cases were ultimately diagnosed as malignant and 2 as benign. Among the benign cases, one was due to chronic pancreatitis, and the other was of unknown etiology. Among the malignant cases, there were 16 perihilar bile duct cancers, 12 distal bile duct cancers, 6 pancreatic ductal adenocarcinoma causing biliary strictures, and one lymph node metastasis from gallbladder cancer causing a biliary stricture.

### 3.2. Specimen Collection and Diagnostic Accuracy

The number of brush cytology attempts was 1 in 26 cases (70.3%), 2 in 6 cases (16.2%), and 3 in 5 cases (13.5%). Biopsies were performed once in 35 patients (94.6%) and twice in 2 patients (5.4%). At our institution, we typically perform brush cytology sampling once and tissue biopsy sampling once and generally conclude the specimen collection procedure when tissue acquisition is macroscopically confirmed. Therefore, we defined the conventional group as cases where brush cytology sampling was performed once, or tissue biopsy sampling was performed once. In the conventional group, the specimen collection rate for brush cytology was 70.3% (26/37), whereas in the B-ROSE group, which allowed for additional sampling, it increased to 89.2% (33/37). Similarly, the biopsy specimen collection rate increased from 94.6% (35/37) in the conventional group to 97.2% (36/37) in the B-ROSE group (Table 2).

Test results were defined as negative when brush cytology or imprint cytology indicated benign findings and positive when they indicated malignant findings. True-negative cases were defined as benign findings confirmed by histopathological results or at six months of follow-up, whereas true-positive cases were confirmed as malignant. Among the 37 cases, there were 12 test-negative cases, 25 test-positive cases, 2 true-negative cases, and 35 true-positive cases. This resulted in a sensitivity, specificity, and diagnostic accuracy of 71.4%, 100%, and 73.0%, respectively (Table 3).

In the conventional group, in which brush cytology was performed only once, the sensitivity, specificity, and diagnostic accuracy were 48.6% (17/35), 50% (1/2), and 48.6% (18/37), respectively. When a biopsy was performed only once, the sensitivity, specificity, and diagnostic accuracy were 68.6% (24/35), 100% (2/2), and 70.3% (26/37), respectively. In the B-ROSE group, which included additional sampling from both brush cytology and biopsy, the sensitivity improved to 71.4%, the specificity remained at 100%, and the diagnostic accuracy increased to 73.0%, demonstrating better results than those of the conventional group.

The diagnostic accuracy of brush cytology significantly improved with additional sampling, whereas biopsy procedures showed no incremental benefit. For brush cytology, the diagnostic accuracy increased from 11/37 cases (29.7%) after the first procedure to 20/37 cases (54.1%) after the second procedure, representing a 24.3% absolute improvement. McNemar’s test demonstrated a statistically significant improvement in diagnostic accuracy with the additional brush cytology procedure (*p* = 0.003). In contrast, the diagnostic accuracy of biopsy remained unchanged at 23/37 cases (62.2%) for both the first and second procedures.

### 3.3. Adverse Events

Adverse events following ERCP occurred in four patients, none of which were associated with multiple sampling. All adverse events were resolved with conservative treatment (Table 4).

## 4. Discussion

ERCP remains the cornerstone in diagnosing biliary strictures, offering high spatial resolution for distinguishing benign from malignant conditions and for evaluating the longitudinal extent of bile duct cancer [14,15,19]. Through bile cytology, brush cytology, and fluoroscopy-guided biopsy, ERCP provides a minimally invasive method of tissue acquisition. However, the diagnostic sensitivity of these techniques remains suboptimal, with reported sensitivities of approximately 30% for bile cytology, 41.4% to 45% for brush cytology, and 48% to 53.4% for biopsy [4,20,21,22,23,24,25].

In this prospective pilot study, the application of B-ROSE resulted in improved diagnostic performance. The overall sensitivity, specificity, and accuracy of B-ROSE were 71.4%, 100%, and 73.0%, respectively, which compare favorably to conventional single-sampling approaches. The ability to immediately assess specimen adequacy during ERCP contributed to higher sampling success rates—particularly for brush cytology, which increased from 70.3% in the conventional group to 89.2% in the B-ROSE group. Similarly, the adequacy of biopsy specimens improved from 94.6% to 97.2%.

To contextualize our results, we compared our findings with the seminal systematic review and meta-analysis conducted by Navaneethan et al., which evaluated the diagnostic performance of conventional endoscopic sampling techniques for malignant biliary strictures [4]. Their pooled analysis revealed that brush cytology achieved a sensitivity of 45% (95% CI, 40–50%), while intraductal biopsy demonstrated a marginally higher sensitivity of 48.1% (95% CI, 42.8–53.4%). When these traditional techniques were used in combination, the diagnostic sensitivity improved only modestly to 59.4% (95% CI, 53.7–64.8%), highlighting the inherent limitations of conventional sampling methods. In contrast, our B-ROSE-enhanced biopsy technique achieved a sensitivity of 71.4%, representing a clinically meaningful improvement of 12 percentage points over the best-performing combination of traditional methods reported in their meta-analysis.

Our results showed that additional brush cytology significantly improved diagnostic accuracy from 29.7% to 54.1% (*p* = 0.003), while repeated biopsy provided no incremental benefit, maintaining 62.2% accuracy. The addition of a second biopsy procedure did not result in any additional correct diagnoses, indicating no incremental diagnostic value. These findings suggest that while repeated brush cytology procedures provide significant diagnostic benefit, additional biopsy procedures did not substantially contribute to improving diagnostic accuracy. 

Notably, B-ROSE not only enhances diagnostic performance but also provides a framework to reconsider procedural strategies during ERCP. Real-time feedback enables clinicians to perform additional sampling as needed, thereby reducing the risk of false-negative results and minimizing the need for repeat procedures. This is particularly beneficial in reducing patient burden, procedural time, and healthcare costs. In cases where B-ROSE confirms specimen adequacy, additional invasive procedures—such as EUS-FNA—may be avoided, streamlining the diagnostic workflow within a single session. At our institution, brush cytology was conventionally performed only once in most cases. However, with B-ROSE in this study, we were able to make real-time decisions to perform additional brush cytology in 11 of 37 cases (29.7%). Among these 11 cases with additional brush cytology, 9 cases achieved accurate diagnosis through brush cytology. This may have prevented the need for a second ERCP or additional examinations when initial diagnosis was inconclusive.

However, diagnostic accuracy varied depending on the etiology of the malignant biliary stricture. The accuracy was highest in distal bile duct cancer (81.3%) and perihilar bile duct cancer (75.0%) but was notably lower for strictures caused by pancreatic cancer (50%) and gallbladder cancer with lymph node metastasis (0%). These discrepancies likely reflect differences in tumor biology and the degree of direct involvement of the bile duct epithelium. In such cases, EUS-FNA remains a valuable adjunctive modality, although its use must be carefully weighed against potential risks such as bile leakage and tumor seeding. When ERCP has difficulty differentiating between benign and malignant causes of biliary strictures, or when specimen collection is challenging with ERCP, we sometimes perform EUS-FNA for specimen collection at our institution. When performing EUS-FNA, similar to B-ROSE cases, a cytotechnologist is present in the endoscopy room to conduct ROSE. By confirming whether adequate specimens have been obtained, we can perform the procedure with a minimal number of needle passes. When ROSE indicates inadequate specimen collection, it also helps in deciding whether to change the type of needle.

While B-ROSE provides a promising enhancement to the ERCP diagnostic arsenal, its broader clinical implementation may face practical challenges. The method relies on the availability of trained cytotechnologists for immediate slide interpretation, which may not be feasible in all institutions. The integration of artificial intelligence-assisted cytology for real-time slide evaluation could expand accessibility and standardize interpretations across centers.

This study also raises the need for further investigation into the long-term clinical implications of B-ROSE. Although we demonstrated improved diagnostic accuracy within a six-month observation period, the effect of early and accurate diagnosis on treatment initiation, patient prognosis, and survival remains to be elucidated. Moreover, cost-effectiveness analyses are warranted to determine whether the initial resource investment in B-ROSE is offset by reductions in repeat procedures, hospitalizations, or advanced imaging.

A major limitation of this study is its small sample size and single-center design, which may limit the generalizability of the findings. Future multicenter prospective trials with larger and more diverse patient populations are necessary to validate our results. Subgroup analyses focusing on rare malignancies, benign strictures, and various anatomical locations will further clarify the role of B-ROSE in clinical practice.

In conclusion, B-ROSE improves the diagnostic accuracy of ERCP-guided sampling for biliary strictures, particularly by enhancing specimen adequacy and reducing the need for repeat interventions. While additional studies are needed to confirm these findings and assess broader clinical impact, B-ROSE holds promise as a practical and effective adjunct to conventional ERCP-based diagnosis.

## Figures and Tables

**Table 1:** Patient characteristics and final diagnoses.

Groups	
Number of patients	37
Age, median (IQR), years	73(70–78.5)
Sex (male/female)	27/10
ECOG-PS (0/1/2)	(27/9/1)
Antithrombotic drug	5
Bile duct stricture (distal/portal)	19/18
Final Diagnosis	Malignant: 35
	Perihilar bile duct cancer: 16
	Distal bile duct cancer: 12
	Biliary stricture due to pancreatic ductal adenocarcinoma: 6
	Biliary stricture due to lymph node metastasis of gallbladder cancer: 1
	Benign: 2
	Chronic pancreatitis: 1
	Unknown etiology: 1

IQR, interquartile ranges; ECOG, eastern cooperative oncology group; PS, performance status.

**Table 2:** Specimen collection and sampling attempts.

Method	Number of Attempts	Specimen Collection Rate
Brush Cytology	1 Attempt: 26	Conventional: 70.3%
	2 Attempts: 6	B-ROSE: 89.2%
	3 Attempts: 5	
Biopsy	1 Attempt: 35	Conventional: 94.6%
	2 Attempts: 2	B-ROSE: 97.2%

**Table 3:** Sensitivity, specificity, and accuracy.

Group	Sensitivity	Specificity	Accuracy
Brush Cytology	48.6%	50.0%	48.6%
Biopsy	68.6%	100.0%	70.3%
B-ROSE (Combined)	71.4%	100.0%	73.0%

**Table 4:** Adverse events.

Age	Sex	Papilla	Biliary Stricture	Brush Cytology (Times)	Biopsy (Times)	Final Diagnosis	Adverse Events	Therapy
76	F	Post EST	Distal	1	1	Bile duct cancer	Hyperamylasemia	Conservative
77	M	Post EST	Distal	1	1	Bile duct cancer	Hyperamylasemia	Conservative
78	F	Intact	Distal	1	1	Pancreatic cancer	Pancreatitis (moderate)	Conservative
73	M	Post EST	Distal	1	1	Chronic pancreatitis	Pancreatitis (mild)	Conservative

F, female; M, male; EST, endoscopic sphincterotomy.

## Data Availability

The datasets used and/or analyzed in the current study are available from the corresponding author upon reasonable request.

## Abbreviations

ERCP	endoscopic retrograde cholangiopancreatography
ROSE	rapid on-site evaluation
EUS-FNA	endoscopic ultrasound-guided fine needle aspiration
B-ROSE	brush/biopsy rapid on-site evaluation
POCS	peroral cholangioscopy
ENBD	endoscopic nasobiliary drainage
ROSE-TIC	ROSE for touch imprint cytology
EST	endoscopic sphincterotomy
